# Use of dual energy CT urography in evaluation of urinary stone and complex cyst

**DOI:** 10.55730/1300-0144.5581

**Published:** 2022-10-22

**Authors:** Mehmet GEZER, Leyla KARACA, Zeynep MARAŞ ÖZDEMİR, Ayşegül KAHRAMAN, Fatih OĞUZ, Fatih ERBAY, Hüseyin YETİŞ

**Affiliations:** 1 Department of Radiology, Faculty of Medicine, İnönü University, Malatya, Turkey; 2 Department of Urology, Faculty of Medicine, İnönü University, Malatya, Turkey

**Keywords:** Dual energy, CT urography, urinary system stones, cyst

## Abstract

**Background/aim:**

Dual-energy computed tomography scans can provide significant benefits to the urinary system. The aim of this study is to determine the limitations and benefits of using dual energy CT urography in patients with urinary system stones and cysts.

**Materials and methods:**

In the analysis of the images, the virtual noncontrasted images obtained from the combined nephrogenic-excretory phase and the true noncontrasted images were evaluated. The true noncontrast images were accepted as the gold standard for stone detection.

**Results:**

Eighty-three different stones were detected in 26 of the 115 patients included in the study. Sensibilities of virtual noncontrast images in detecting urinary system stones were 66.7% and 65.4% according to the first and second radiologists, respectively.

In this study, 32 hyperdense cysts were detected. According to iodine map images, there was no enhancement in 26 of 32 cysts; only 5 cysts showed minimal contrast enhancement. One patient could not decide on contrast enhancement.

**Conclusion:**

As a result, if CT urography is performed with dual energy, it can provide additional information in patients with urinary system disorder.

## 1. Introduction

The development of computed tomography from single-detector scanner systems to multidetector scanner systems has significantly affected the radiologic assessment of the urinary tract [1]. CT urography provides detailed and comprehensive evaluation of the urinary tract. The radiation dose in CT urography can be reduced with dual energy CT by reducing the number of imaging phases or by the split bolus technique [2,3]. Dual energy CT can be used for mass characterization in various systems such as the abdomen, lung, cardiac and central nervous system. It can also be used in the urinary system for stone characterization and evaluation of complicated cyst masses [4–6]. DECT scans can provide significant benefits to the urinary system. With dual energy, virtual noncontrast images can be obtained and lesion characterization can be better. According to conventional single-energy CT examination for characterization of kidney masses, the most important advantage of DEBT examinations is that it provides valuable information with contrast-enhanced scans about the lesion without any additional noncontrast scans [7–10].

The use of DECT urography in urinary stone disease may potentially be detection of stones with virtual noncontrast images obtained by DECT, without need of true noncontrast scan [11,12]. In general, the virtual noncontrast images obtained from the excretory phase have a accuracy of 53%–87% in stone detection [13,14]. The factors affecting the determination of stone include stone size (small stones are harder to detect) and density (low density of stones is more difficult to detect) [12–14.]. The most common disorders in CT urography examinations are stones and cysts.

In this study, we aimed to the detectability of urinary system stones and complicated cysts in virtual non contrast images using DECT. In light of these findings, we aim to determine the advantages and weaknesses of DECT.

## 2. Materials and methods

### 2.1. Patient population

One hundred and fifteen patients who underwent DECT urography between 2017 and 2020 were evaluated. The Institutional Review Board approved this prospective study. Written informed consent of all patients was obtained. Routine multiphase CT urography is performed in our hospital, and we planned to perform split bolus CT urography with DECT on the patients who were included in the study.

Male and female patients over age of 18 were included in the study. Patients under age of 18, pregnant patients and patients with suspected pregnancy were excluded from the study. In one patient, precontrast images could not be optimally obtained and removed from the study because the patient had received contrast agent for another reason before the shooting. In addition, in another patient, the parenchymal calcification foci could not be clearly distinguished from the stones and removed from the statistical analysis. Nephrocalcinosis was present in precontrast images of one patient. Nephrocalcinosis could not be differentiated in virtual noncontrast images. In addition, cystic renal masses were excluded because they were not the subject of current study.

### 2.2. CT protocol

In this study, a dual source DECT (Siemens Healthcare, Forchheim, Germany) device was used and the images were evaluated by sending them to Siemens Syngo. Via Workstation. At a flow rate of 4 mL/s, 40% of the contrast agent (Iohexol-omnipaque 350 mg/mL) determined according to the patient’s weight (1.5 mg/kg) was administered. Our purpose was to fill the excretory system with contrast. 15 min later, the remaining 60% was delivered at a flow rate of 4mL/s and, after 75–90 s, combined nephrogenic-excretory phase images were taken using dual energy mode. In the protocol of our study, the precontrast phase parameters were: detector 128 × 0.6, pitch: 0.5, rotation time: 0.5 s, slice thickness: 1.5 mm, matrix: 512 × 512; in the postcontrast phase parameters were: detector 128 × 0.6, pitch: 0.6, turn time: 0.5 s, section thickness: 1.5 mm, scanning using matrix: 512 × 512. Tube voltages were set to tube A 100 kV and tube B to 140 kV.

### 2.3. Analysis of images

All images were examined by two radiologists with 4 and 20 years of radiologic experience. In the analysis of the images, the two radiologists first examined virtual noncontrast images and real noncontrast images independently. Virtual noncontrast images were obtained from the combined nephrogenic-excretory images using the Liver VNC (Virtual noncontrast) algorithm in the Syngo. When a urinary system stone was detected in one patient, the localization of the stone was noted by dividing the urinary system into 4 anatomical segments (1, precalyceal or calyceal; 2, pelvis; 3, ureter; 4, bladder). The diameters of the stones were measured independently by both radiologists on both virtual and true noncontrast images. In the virtual noncontrast images, classification was made as stone present or not in the urinary tract. True noncontrast images were considered the gold standard for the presence of stones (Figures 1A–1C). The images were evaluated in the axial plane and in the multiplanar format by coronal and sagittal reconstruction if the radiologist deemed it necessary. However, diameter measurements were made from the axial slice with the largest stone diameter. The default values and bone window presets in the Liver VNC algorithm were used for examining images and measuring stone diameters (Figures 2A–2C). However, both radiologists were allowed to adjust the window settings as desired. In the case of multiple stones in the same patient, the proximal stone was always measured first. True noncontrast images were examined by both radiologists (especially in patients with multiple stones) to confirm that the measurements were from the same stones.

In addition, both radiologists identified complicated-hyperdense cysts with consensus (Figures 3A–3C). Dual Energy software was used for postcontrast combined nephrogenic-excretory phase images. In the iodine map, color coding was accepted as a form of contrast enhancement. When iodine map images of these cysts were examined, 26 of 32 hyperdense cysts did not show contrast enhancement, whereas mild color coding detected in 5 cysts was evaluated in favor of minimal contrast enhancement. On iodine map imaging, enhancement was considered present with a visual impression of color areas concessus. No decision could be made about contrast enhancement of only one cyst.

### 2.4. Statistical analysis of data

According to the power analysis, the minimum sample size required to determine the sensitivity of virtual noncontrast images with a tolerance value of 0.05 at 95% confidence level was calculated as 81. Data was summarized as mean ± standard deviation and/or median (min-max). The suitability of the data to the normal distribution was examined by the Kolmogorov-Smirnov test. Wilcoxon test was used for variables that did not show normal distribution, and t-test was used for paired samples. p <0.05 was considered statistically significant. Categorical data were analyzed by Pearson correlation test (p <0.01). For data analysis, the IBM SPSS Statistics Version 22.0 package program was used.

## 3. Results

In our study of the 115 patients who underwent DEBT urography, 78 (67.8%) of them were male, and 37 (32.2%) of them were female. According to the true noncontrast images, 83 different stones were detected in 26 patients. Of the 26 patients who had stones, 20 (76.9%) were male and 6 (23.1%) were female.

In the study, 32 complicated cysts were detected with the consensus of two radiologists. Of the 32 patients with complicated cysts, 29 (90.6%) were male and 3 (9.3%) were female. There was no enhancement in 26 of 32 cysts; only 5 cysts showed minimal contrast enhancement. One patient could not decide on contrast enhancement.

### 3.1. Measurement and analysis of patients with stone

Of the 115 patients included in the study, 83 different stones were detected in 26 patients. Both researchers evaluated the two stones located close to each other as one stone in virtual noncontrast images. In the statistical analysis of the data, these stones were not included in the evaluation.

Evaluation of the researcher 1 (4 years of radiology experience); 3 hyperdense foci evaluated as stones in virtual noncontrast images could not be confirmed in the true noncontrast images and they were considered as false positives. In virtual noncontrast images, 54 of the 81 stones detected in true noncontrast images were also detected. Of those, 27 could not be detected on virtual noncontrast images. Sensitivity of virtual noncontrast images obtained from the combined nephrogenic-excretory phase was 66.7% (54 of 81 stones).

According to the measurements of the first researcher, the mean diameter of 81 stones in the true noncontrast images was 7.04 mm (1–23.4 mm; standard deviation = 5.1). In virtual noncontrast images, the mean diameter of the stones that could not be detected was calculated as 3.39 mm. There were 54 stones detected in virtual noncontrast images. The mean diameter of those stones in the true noncontrast- and virtual noncontrast series was 8.87 and 8.64, respectively. No statistically significant difference was found in the statistical comparison of the largest diameters of these stones in virtual non contrast and true noncontrasted images (p = 0.276).

Evaluation of the researcher 2 (20 years of radiology experience); one hyperdense focus evaluated as stone in virtual noncontrast images could not be confirmed in the true noncontrast images and it was considered a false positive. Fifty-three of the 81 stones detected in true noncontrast images were also detected in virtual noncontrast images. Of those, 28 could not be detected on virtual noncontrast images. Sensitivity of virtual noncontrast images obtained from the combined nephrogenic-excretory phase was 65.4 % (53 of 81 stones).

According to the measurements of the second researcher, the median diameter of 81 stones in the true noncontrast images was 5.9 and the mean diameter was 7.29 mm.

The mean diameter of the stones, which could not be detected in virtual noncontrast images, was calculated as 3.6 mm. 53 stones were detected in virtual noncontrast images. The median and mean diameters of those stones in true noncontrast images were 7.8 mm and 9.23 mm, respectively. The median and mean diameters of those stones in virtual noncontrast images were 7.7 mm and 8.53 mm, respectively. A statistically significant difference was found that to comparison of the largest diameters of these stones in virtual noncontrast and true noncontrasted images (p = 0.005).

**Figure 4:**
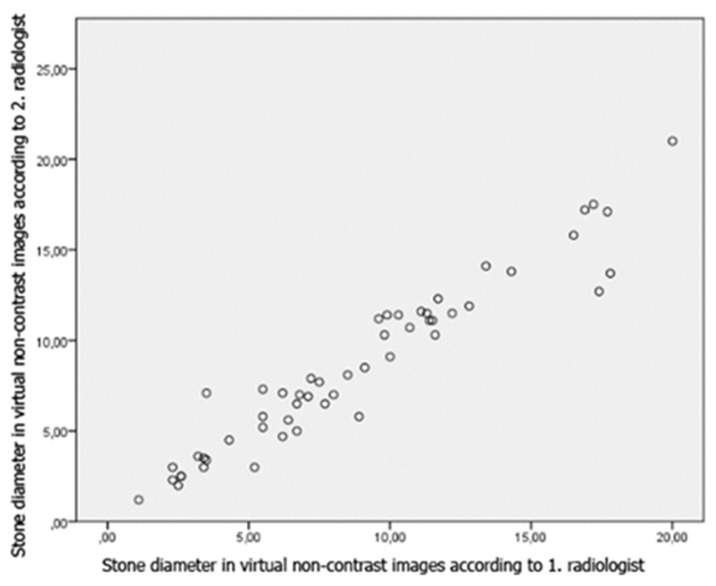
Pearson correlation of both researcher’s stone diameter measurements to each other on virtual noncontrast images.

**Figure 5:**
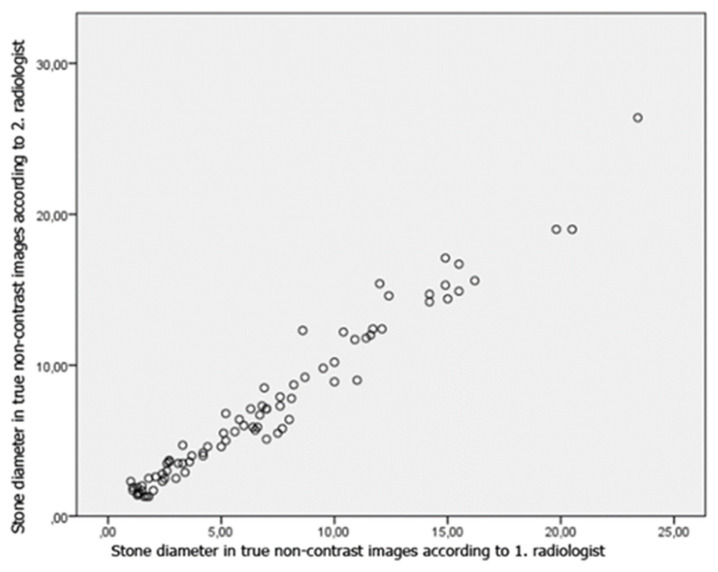
Pearson correlation of both researcher’s stone diameter measurements to each other on true noncontrast images.

Pearson correlations of two researchers in terms of stone diameters in true and virtual noncontrast images were high (0.980, p < 0.001). The correlation of the measurements of the researchers with each other are shown in Figures 4 and 5.

### 3.2. Analysis of patients with complicated cysts

**Figure 6:**
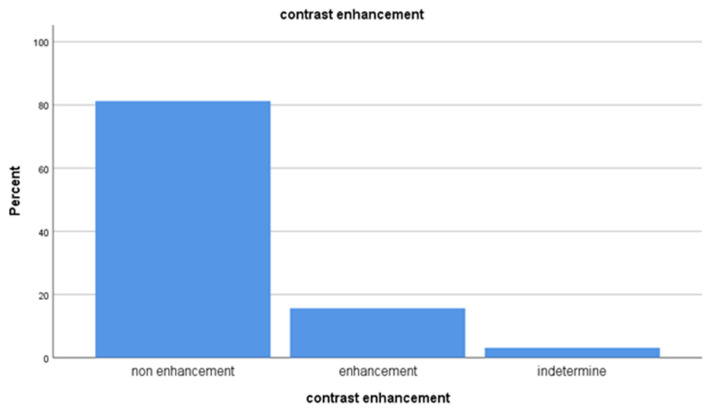
The figure shows the distribution frequency of complicated cysts on the iodine map.

In the study, 32 of 115 patients included had 32 complicated cysts with the consensus of both radiologists. When iodine map images of these cysts were examined, 81.3% of the complicated cysts did not show contrast enhancement (Figures 3A–3C). Minimal contrast enhancement was detected in 15.6% of the cysts (Figure 6). Contrast uptake could not be evaluated in only 3.1% of the cysts. There was no significant difference in evaluation between the two researchers. (Z= –1.414, p = 0.157). The two researcher interpreted the absence of contrast enhancement with 95% confidence interval.

## 4. Discussion

CT urography is an excellent technique to show the stones in the urinary tract. Takahashi et al. found a 63% (23 of 43 stones) stone detection sensitivity of the virtual noncontrast images obtained from the pyelogram phase [14]. Scheffel et al. found stone detection sensitivity as 74% (26 out of 35 stones) in the virtual noncontrast images obtained from the nephrogram phase [15]. However, in this study, the sensitivity of the original images in the nephrogram phase (without iodine removal) was found to be 94%. As a result, compared to the original image, virtual noncontrast images could not contribute to the detection of urinary tract stones, according to this study. In a study by Bosticas et al., they found the sensitivity to be 78% (18 out of 23 stones) in virtual noncontrast series obtained after DECT urography following furosemide injection [16]. However, in this study, similar to the study of Scheffel et al. sensitivity was higher (86%) in the original images (without iodine removal). In addition, the use of furosemide in this study may have increased the detectability of stones [17].

In our study, virtual noncontrast images were obtained from combined nephrogenic-excretory images. The sensitivity of detecting urinary system stones in virtual noncontrast images was calculated as 66.4% and 65.4% according to the first and second radiologists, respectively. In our study, the sensitivity of the virtual noncontrast images for detecting urinary tract stones was similar to that in the literature. In the study of Botsicas et al. the higher sensitivity than in our study may be due to the fact that in the study they used furosemide injection [16]. We did not use furosemide in our study. In addition, in our study, according to the first radiologist, 3 hyperdense foci and according to the second radiologist one hyperdense focus in the virtual noncontrasted images could not be verified in true noncontrast series. It could be that iodine could not be fully subtracted by the algorithm and the remaining iodine signal was evaluated as stone by the researcher. We think of this as a weakness of dual energy software algorithms. In addition, both researchers evaluated the two stones, which were observed in the bladder close to each other in the true noncontrast images, as a single stone in the virtual noncontrast images. The reason for this may be the excess iodine concentration in the bladder in the combined nephrogenic-excretory phase and the inability of the iodine signal to be optimally removed during the subtraction.

Takahashi et al. reported in their study that the sizes of most stones were slightly smaller on virtual noncontrast images when compared to the true noncontrast series [14]. They stated the reason for this is that the signal in the periphery of the stone might be slightly substracted by the iodine during the generation of virtual noncontrasted images. Moon et al. found in a study with 80 stones in 32 different patients that the size of the stone measured in true noncontrasted images was statistically significantly greater than that of virtual noncontrast images obtained from nephrogram and excretory phases [12]. They stated that this might be due to partial removal of stone signal with iodine signal during substraction.

In our study, the correlation between the two radiologists was found to be high, in terms of stone diameters measured in the true and virtual noncontrast images (Figures 4 and 5). However, a statistically significant difference was found between the stone diameters measured in the virtual and true noncontrast images, according to the second radiologist. This may be due to the difference in measurement between radiologists, suboptimal iodine subtraction by the postprocessing algorithms or, as Takahashi et al. stated, partial removal of stone signal with iodine signal during subtraction [14]. In a study performed by Karlo et al., they found the mean diameter of the stones which could not be detected in the virtual noncontrast images obtained from the combined nephrogenic-excretory phase as 2. 5 mm [17].

In our study, the mean diameters of the stones were similar according to the first and second radiologists. They are smaller than the diameters of the stones detected in the virtual noncontrast images. Since stones detected in virtual noncontrast images obtained by dual energy CT urography are greater in size than the undetectable stones, dual energy CT urography can show clinically meaningful stones and can be useful in urinary tract stone disease. However, since small stones may be clinically the cause of hematuria and these stones are more likely to be undetectable in virtual noncontrast images, virtual noncontrast images may not be as useful as true noncontrast images in terms of stone detection. In addition, in our study, a caliceal stone with an approximate diameter of 15 mm was not detected by two researchers. This may be due to the excess amount of contrast in the calyx and insufficient subtraction of iodine by the algorithm.

The majority of cysts detected in the kidney are simple cysts, and their densities are close to the density of the water. Cysts with higher densities have hemorrhagic or proteinous content. Since these cysts may be hyperdense in precontrast images, it may be difficult to determine whether they have enhanced in postcontrast images. These can be confused with malignant lesions such as renal cell carcinoma. Such lesions may be characterized as malignant or benign, depending on their contrast enhancement. Some radiologists evaluate contrast enhancement as a 10 HU density increase in postcontrast images. Some radiologists accept a 20 HU density increase as contrast enhancement [18,19]. Iodine maps DECT can be used to examine contrast enhancement. Brown et al. reported that the color-coded iodine map technique was sensitive in demonstrating contrast enhancement in their study using a phantom model [19]. L’Hostis et al. reported that iodine concentration maps with DECT perform as well as improved images obtained with conventional CT in the detection and characterization of tissue and atypical cystic kidney lesions [20]. They quantitatively evaluated contrast enhancement. In our study, we compared the differences (postcontrast-precontrast) with the visual color coding in the iodine map. Iodine map images can help us to show whether these lesions are enhancing or not. In our study, although the number of enhancement cysts was low, it was found that iodine map images could be useful in excluding contrast enhancement of complex cysts when compared with density difference. DECT can determine with (81.3%) high accuracy that complex cysts do not enhance contrast. Therefore, we think that dual energy should be used in routine examination because it will allow the evaluation of the contrast enhancement of both complicated cysts and masses.

In this context, besides small stones, virtual noncontrast images obtained from the combined nephrogenic-excretory phase may not detect large stones that may be clinically important. However, dual-energy CT urography may provide additional benefit to the patient by characterization of stones and by showing lesion enhancement with iodine map images. Therefore, we recommend that CT urography examinations be performed with DECT. It also gives researchers the opportunity to do this without overdosing.

There are some limitations to our study. In our study, we created virtual noncontrast images only from the combined nephrogenic-excretory phase images. The results could be different if multiphase CT urography was performed and virtual noncontrast images were created from other phases and compared with each other or true noncontrast images. As the number of samples in our study is low, different results can be obtained in a larger series. Complicated cysts were evaluated only qualitatively in our study. Quantitative evaluation will yield more valuable results. In addition, personal interpretation differences between radiologists may have caused the measurements to be different.

DECT urography can detect urinary system stones, but due to some limitations and technical features, they cannot show all stones as true noncontrasted images. However, it provides additional information that cannot be obtained with true noncontrast images. In addition, although different studies were needed, the results of our study showed that iodine map images could be useful in the exclusion of contrast enhancement and thus may help with the characterization of complicated renal cysts.

In conclusion, if CT urography is taken with dual energy, it provides additional information about the patient without causing an increase in dose. However, for virtual noncontrast images to be able to detect urinary system stones as true noncontrast images, there is a need for further development of DECT technology/postprocessing algorithms and more studies on this issue.

## Figures and Tables

**Figure 1A:**
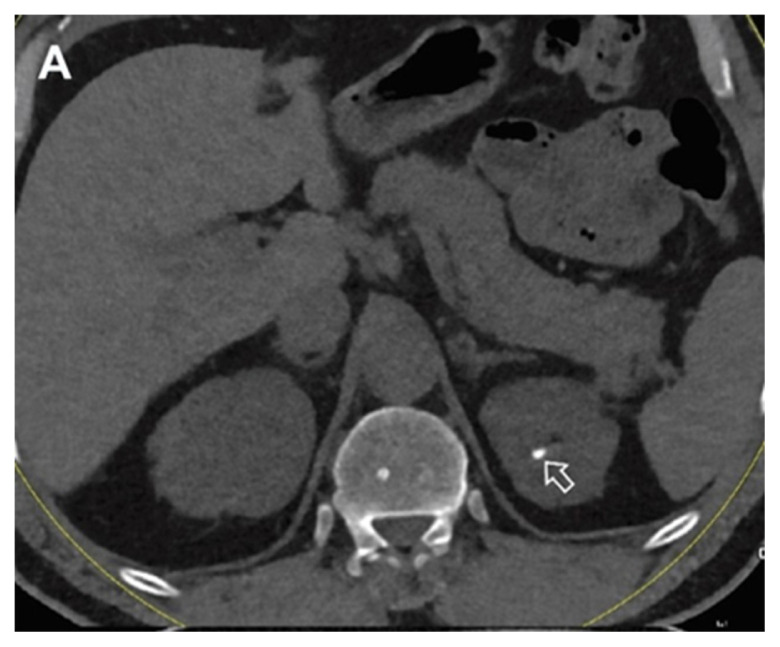
A 48-year-old male patient the virtual noncontrast image obtained from the combined nephrogenic-pyelogram phase in the left kidney stone (arrow).

**Figure 1B:**
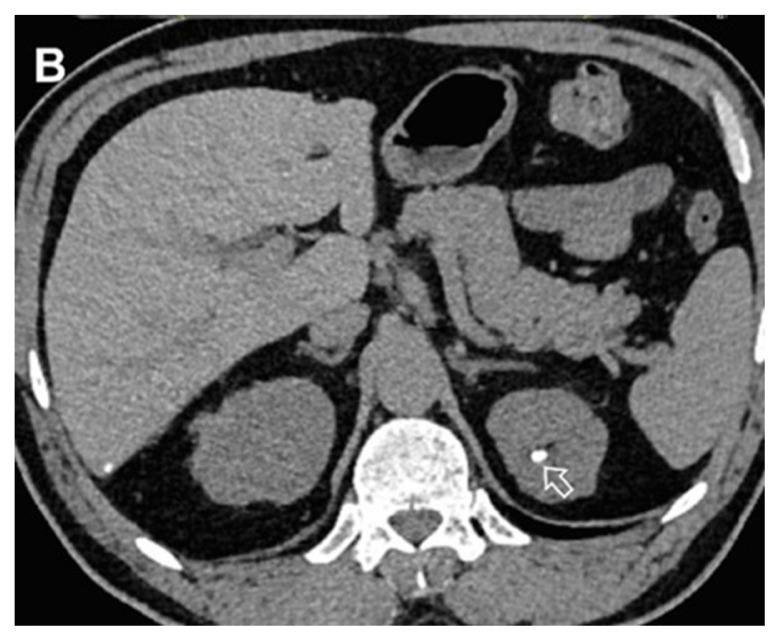
A 48-year-old male patient with true noncontrast image in the left kidney stone (arrow).

**Figure 1C:**
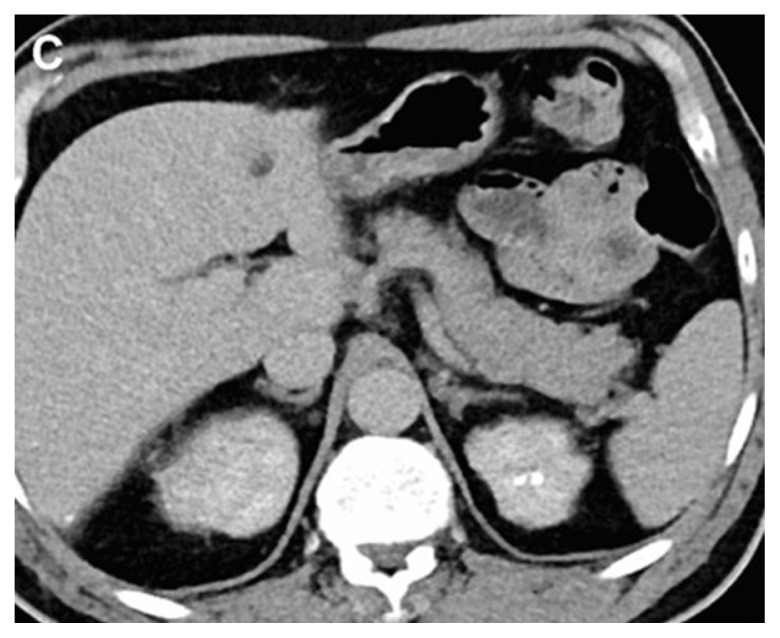
A 48-year-old male patient the image combined nephrogenic-pyelogram phase in the left kidney stone.

**Figure 2A:**
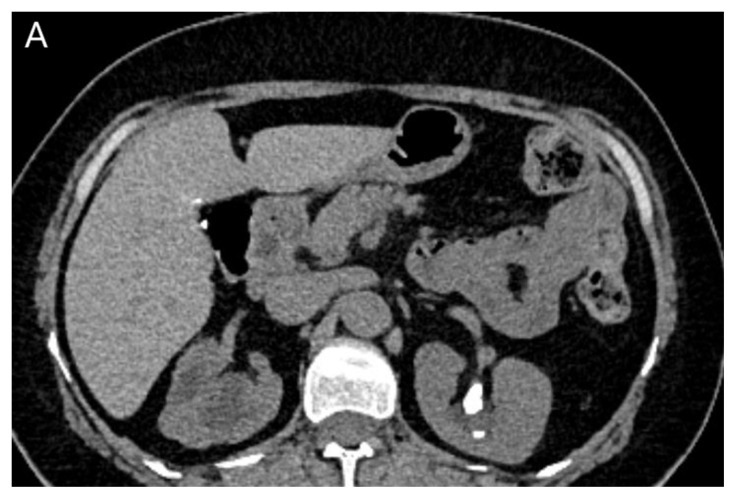
In a 41-year-old female, left kidney stones are clearly seen in true noncontrast images.

**Figure 2B:**
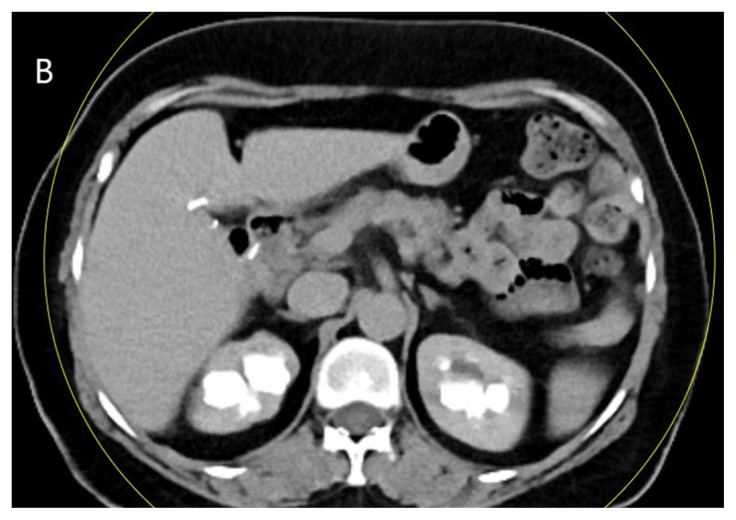
The postcontrast combined nephrogenic-pyelogram phase.

**Figure 2C:**
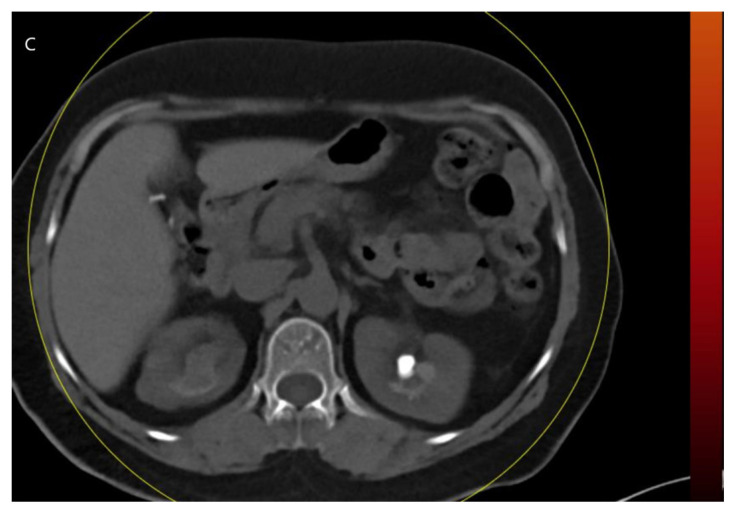
In a 41-year-old woman, virtual noncontrast images show the small stone indistinguishable from the iodine signal.

**Figure 3A:**
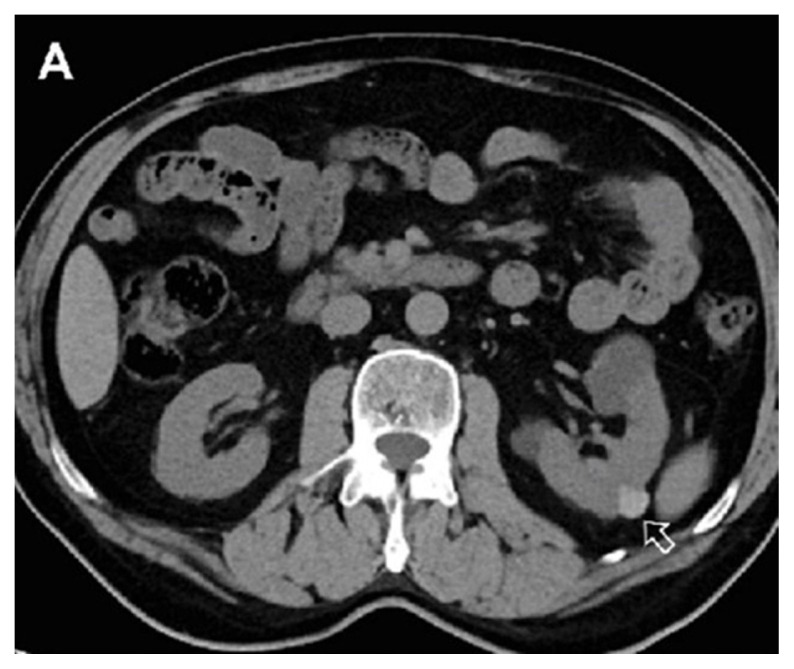
In a 62-year-old male patient, hyperdense cyst in the left kidney (arrow) is seen in true noncontrast images.

**Figure 3B:**
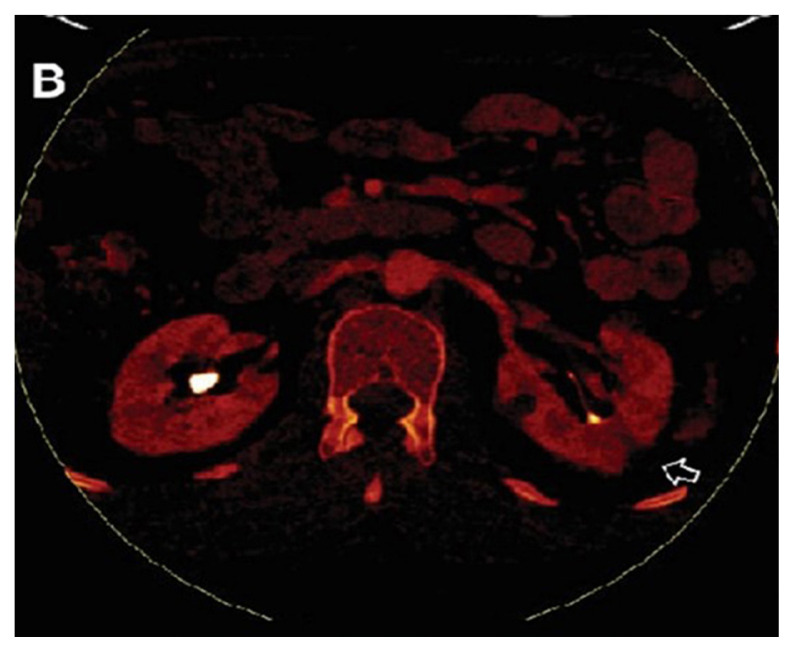
In the iodine map images, the cyst does not show contrast enhancement.

**Figure 3C:**
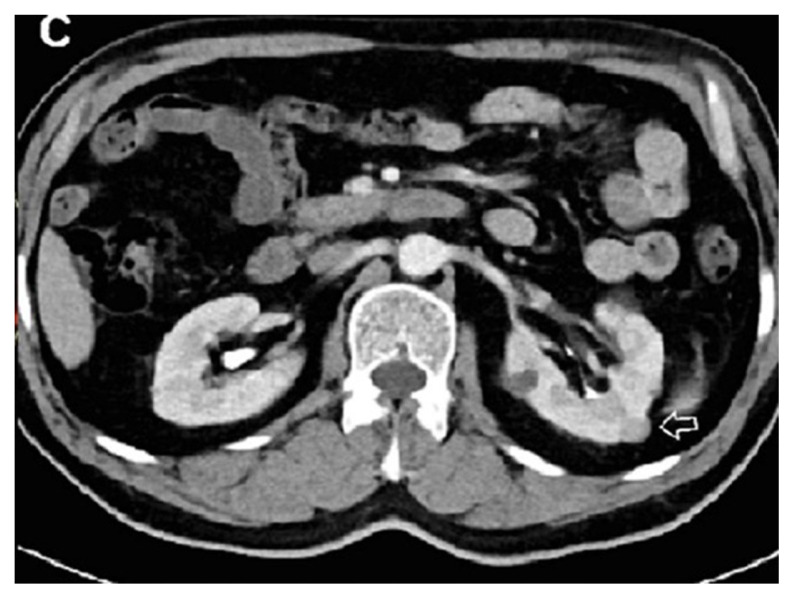
In the combined nephrogenic-pyelogram phase image, it is difficult to evaluate the contrast enhancement since the cyst is hyperdense (arrow).
